# How to Balance Prognostic Factors in Controlled Phase II Trials: Stratified Permuted Block Randomization or Minimization? An Analysis of Clinical Trials in Digestive Oncology

**DOI:** 10.3390/curroncol31060259

**Published:** 2024-06-17

**Authors:** Elodie Martin, Karine Le Malicot, Catherine Guérin-Charbonnel, François Bocquet, Olivier Bouché, Anthony Turpin, Thomas Aparicio, Jean-Louis Legoux, Laetitia Dahan, Julien Taieb, Côme Lepage, Louis-Marie Dourthe, Caroline Pétorin, Vincent Bourgeois, Jean-Luc Raoul, Valérie Seegers

**Affiliations:** 1 Institut de Cancérologie de l’Ouest, F 49055 Angers, France; 2 Fédération Francophone de Cancérologie Digestive (FFCD), EPICAD INSERM LNC-UMR 1231, University of Burgundy, F 21078 Dijon, France; 3 CRCI2NA, INSERM U1307, CNRS UMR6075, University of Nantes, F 44000 Nantes, France; 4 Law and Social Change Laboratory, Faculty of Law and Political Sciences, CNRS UMR 6297, Nantes University, F 44035 Nantes, France; 5 Department of Digestive Oncology, CHU Reims, F 51092 Reims, France; 6 Department of Medical Oncology, University Hospital, F 59000 Lille, France; anthony.turpin@chru-lille.fr; 7 Department of Gastroenterology, Saint Louis Hospital, APHP, Université Paris Cité, F 75010 Paris, France; 8 Department of Hepato-Gastroenterology and Digestive Oncology, Centre Hospitalier Régional, F 45100 Orléans, France; 9 C.H.U. la Timone and Université de la Méditerranée Marseille, F 13005 Marseille, France; 10 Institut du Cancer Paris CARPEM, Gastroenterology and Digestive Oncology Department, APHP Centre–Université Paris Cité, Hôpital Européen G. Pompidou, F 75015 Paris, France; 11 Centre de Recherche des Cordeliers, INSERM, CNRS, Sorbonne Université, USPC, Université de Paris, Université Paris Cité, F 75006 Paris, France; 12 Department of HGE & Digestive Oncology, EPICAD INSERM UMR LNC 1231, University Hospital Dijon, University of Burgundy, F 21078 Dijon, France; 13 Service d’Oncologie Médicale, Clinique St Anne, F 67000 Strasbourg, France; 14 Service d’Oncologie Digestive, CHU Clermont-Ferrand, F 63000 Clermont-Ferrand, France; 15 Service d’Oncologie Digestive, Centre Hospitalier de Boulogne sur Mer, F 62321 Boulogne-sur-Mer, France

**Keywords:** minimization, randomization, phase II trials

## Abstract

In controlled phase II trials, major prognostic factors need to be well balanced between arms. The main procedures used are SPBR (Stratified Permuted Block Randomization) and minimization. First, we provide a systematic review of the treatment allocation procedure used in gastrointestinal oncology controlled phase II trials published in 2019. Second, we performed simulations using data from six phase II studies to measure the impacts of imbalances and bias on the efficacy estimations. From the 40 articles analyzed, all mentioned randomization in both the title and abstract, the median number of patients included was 109, and 77.5% were multicenter. Of the 27 studies that reported at least one stratification variable, 10 included the center as a stratification variable, 10 used minimization, 9 used SBR, and 8 were unspecified. In real data studies, the imbalance increased with the number of centers. The total and marginal imbalances were higher with SBR than with minimization, and the difference increased with the number of centers. The efficiency estimates per arm were close to the original trial estimate in both procedures. Minimization is often used in cases of numerous centers and guarantees better similarity between arms for stratification variables for total and marginal imbalances in phase II trials.

## 1. Introduction

After phase I, which is the safety exploration in clinical oncology research, phase II trials are designed to provide preliminary evidence that a new therapeutic strategy seems to be effective enough to proceed to a phase III study for efficacy comparison with the standard of care. Phase II trials in oncology have typically been single-arm trials, but controlled designs are recommended for assessing the current efficacy in both control (standard of care) and experimental groups in the same setting (diverse cancer parameters, multiple centers if possible, etc.). Ideally, for trials with 1:1 ratio, the distribution of prognostic factors must be similarly balanced to prevent selection bias [1,2]. Randomization, aiming to produce treatment groups in which the distribution of prognostic factors, known and unknown [3,4], is well balanced, introduces randomness into the assignment of treatments to participants [3] but does not always guarantee that the groups will be similar with regard to patient characteristics [5]. 

**Figure 1:**
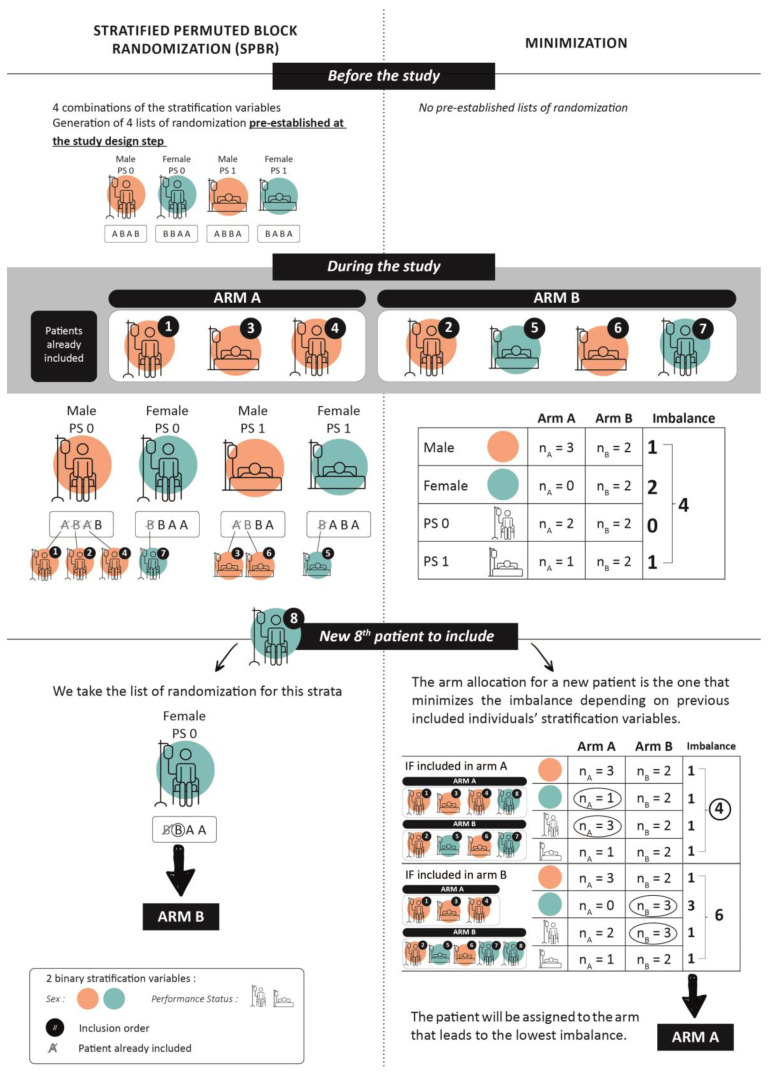
Decision regarding the arm allocation of the 8th patient (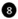) depending on the 2 procedures: Stratified Permuted Block Randomization (**left** side) and minimization (**right** side). The characteristics of the 7 patients who were already randomized in the study are shown at the **top left** of the diagram. The study’s stratification variables are represented using colors and pictograms (see **top right** of diagram).

Initially, simple randomization often led to imbalances in participant groups, particularly in small trials. To address this, block randomization was introduced, ensuring a balance by dividing participants into blocks for random assignment, although it neglected important covariates. Usually, major prognostic factors are known before the study, so stratification was added to balance participants across treatment groups within specific covariates (e.g., smoking status, mutational status). Combining these methods, stratified block randomization allocates participants by key covariates and then applies block randomization within each stratum. The permuted block size in the randomization prevents predictability in treatment assignment, ensuring that the allocation remains truly random while still achieving balanced group sizes, which reduces selection bias and maintains the integrity of the trial. This approach, called “Stratified Permuted Block Randomization” (SPBR), enhances the overall and within-stratum balance, improving the results’ robustness. Before the first patient inclusion (Figure 1), the predefined random sequence for all possible stratifying variables is supposed to ensure a balance in the number of participants between treatment groups in each stratum [6]. If the effect of treatment is expected to vary substantially in magnitude across clinically relevant subgroups, stratifying these subgroups can help demonstrate the treatment’s effect and consistency across these subgroups (Figure 1). In practice, the main limitation of this procedure is that if there are too many strata and/or too few subjects included, many blocks will be incomplete, and an imbalance can occur [7], which may affect the study’s results.

As assignment solely by chance does not guarantee group similarity, other procedures that attempt to balance treatment groups with respect to baseline covariates such as minimization can be used for this purpose [8]. Minimization is a non-random method for treatment allocation, initially proposed by Taves and then by Pocok and Simon [9,10] and validated by Altman [11]. The minimization mechanism lies in assigning the treatment arm to a newly included patient, based on their clinical characteristics, by calculating the differences in these characteristics between arms depending on which arm the patient is assigned to. The arm making the smallest difference (imbalance) possible is chosen (Figure 1). This dynamic adaptive method is designed to reduce differences in the distribution of prognostic factors between treatment group assignments to guarantee the similarity of groups even in small samples [12]. The treatment allocated to the next participant enrolled in the trial depends (wholly or partly) on the characteristics of the participants who are already enrolled (see Figure 1). 

One of the most significant limitations of minimization is that the allocation of the treatment arm is predictable when all the parameters of the patients who were previously included in the study are known. Therefore, it is recommended that the minimization includes some randomness. For example, assuming a 20% randomness factor (also known as a random element), the probability of a patient being oriented towards the most unbalanced arm is, at worst, 20%. This will make the overall procedure difficult to predict, especially in a multicenter trial [13]. In general and in practice, the proportion of randomness chosen varies between 10 and 25% [14,15].

Whatever the allocation treatment method (SPBR or minimization) is in clinical trials, the choice of arm allocation has no impact and is not involved in the sample size calculation, and the European Medicine Agency [16] recommends adjusting the treatment effect for the prognostic variables used for stratification to improve the power.

In this educational manuscript, conjointly written by statisticians and digestive oncologists, we first evaluate the use of these two allocation procedures—minimization and SPBR—in a systematic review of published phase II trials in gastrointestinal oncology, and second, we illustrate the advantages and disadvantages of these procedures, simulating the arm allocation from six of these trials and endpoints from one, using both procedures. 

## 2. Materials and Methods

### 2.1. Literature Search Strategy and Selection Criteria

We conducted a systematic review of controlled phase II clinical trials in a gastrointestinal oncology setting. We limited our search to digestive cancers, as one co-author (JLR) is involved in gastrointestinal oncology and is a member of the FFCD (Fédération Francophone de Cancérologie Digestive) and PRODIGE (Partenariat de Recherche en Oncologie DIGEstive) intergroups. PRODIGE data were chosen because patient-level data can be obtained after a request is made through a committee. We searched MEDLINE (PubMed) for English-language articles to identify controlled phase II trials published between 1 January 2019 and 31 December 2019. Our search algorithm included medical subject heading terms for gastrointestinal neoplasms; the date of publication; and publication type (for a controlled clinical trial, phase II) (the search algorithm can be found in File S1 List S1). We excluded protocol publications, post hoc analyses, historic controls, phase I/II or phase III studies, and single-arm studies. All records retrieved were assessed independently by two authors (EM, VS). First, titles and abstracts were screened to identify obvious exclusions. Second, full-text reports were retrieved to determine whether they met the selection criteria. Any disagreements were resolved through discussion. Data extraction was carried out independently by the two authors (EM and VS) using a pre-designed data extraction form prior to data entry. Information extracted included the following items: the method and parameters used for patient allocation, number of patients, arms, centers, and stratification variables. 

### 2.2. Real Clinical Study Applications

This study received approval by our local ethics committee [Comité d’Ethique CHU d’Angers (2022-096)]. After approval by the FFCD PRODIGE groups, they provided us with anonymized Individual Participant Data from the following 6 published clinical studies: FFCD 9803 [16] (gastric and cardial adenocarcinoma), PRODIGE 16 [17] (hepatocellular carcinoma), PRODIGE 20 [18], PRODIGE 25 [19] (colorectal cancers), and PRODIGE 35 [20] and PRODIGE 37 [21] (pancreatic adenocarcinomas).

#### 2.2.1. Simulating Arm Allocation

From the aforementioned real clinical trial data, we used the individual baseline characteristics that corresponded to the stratification variables described in the protocols of each study [16,17,18,19,20,21] to simulate 1000 allocations of treatment arms using SPBR and minimization successively. The patients’ order of inclusion was re-shuffled for each data set simulation. For the minimization simulation, 20% of randomness was used [13], and for SPBR, the block size was set at twice the number of treatment arms. 

#### 2.2.2. Imbalance Measurements

Imbalance measures the difference between arms for predefined characteristics. We used three levels of precision: total, marginal, and within-stratum imbalances, which were calculated for each allocation method in each of the 1000 simulated data sets in each of the 6 trials.

Table 1 illustrates these three types of imbalances between arms with 2 stratification variables:

1. Total imbalance is the difference measured between arms, calculated using the total number of patients assigned to each arm, 0 in our example.
2. Marginal imbalance (or covariable margin imbalance) is calculated as the sum of the differences between arms for each modality of the variables, 2 in our example.
3. Within-stratum imbalance is calculated as the total differences between treatment arms for each combination of stratification variables, 16 in our example.

#### 2.2.3. Simulation of Endpoints

To explore whether—and how much—the allocation arm method would affect the efficacy estimation, we calculated the efficacy in each arm for each of the 1000 simulations for both methods, and we measured the difference between these estimations and the efficacy measured in the real data from the clinical trial. The PRODIGE 35 and 37 trials [21,22] were chosen to simulate their primary endpoint in relation to the 2 methods studied. The primary outcome of these studies, the 6-month Progression-Free Survival (PFS) rates, was considered a binary variable. The Bernoulli distribution was used to regenerate the endpoint for each patient using the probability of success observed in each stratum. All variables used in the stratification process, except centers, were used to estimate the treatment’s effect in each stratum (any possible combination of stratification variable modalities, e.g., bottom of Table 1). We did not use centers given their high number (52 and 36 centers, respectively) and the small number of patients in each within-stratum (276 patients divided into 624 possible combinations for PRODIGE 35 and 127 patients divided into 432 possible combinations for PRODIGE 37); this did not provide enough information to make a convincing estimate of the efficacy in each stratum. The impact of the allocation arm method on the efficacy estimation was described by calculating the bias on the efficacy estimate, defined as the difference between the actual clinical efficacy obtained in the article and the measured effect in each simulated data set trial. 

### 2.3. Software

All simulations were performed using the R version 4.2.2 (2022-10-31 ucrt): A language and environment for statistical computing (R Foundation for Statistical Computing, Vienna, Austria. URL https://www.R-project.org/ accessed on 5 September 2023). 

For studies with two arms, the PocSimMIN function was used to mimic the minimization procedure and StrPBR for stratified block randomization (SPBR), both of which are available in the carat [15] package. For studies with 3 arms, the Minirand [23] and blockrand [24] packages were used.

## 3. Results

### 3.1. Review of Our Selected Articles

**Figure 2:**
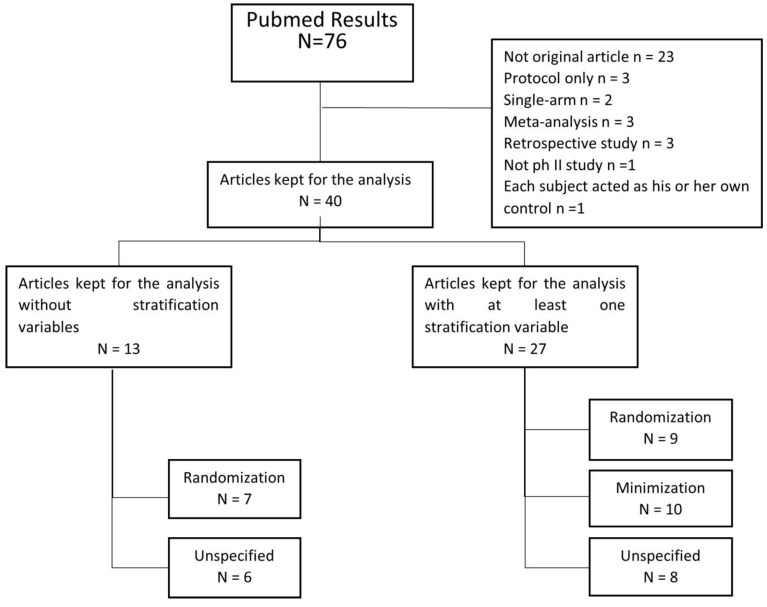
Literature review flowchart: PRISMA flow diagram of our systematic analysis of controlled phase II trials in digestive oncology published in 2019.

The PRISMA flow diagram of the selected articles is presented in Figure 2. After reconciliation, 40 articles were included in the analyses [25,26,27,28,29,30,31,32,33,34,35,36,37,38,39,40,41,42,43,44,45,46,47,48,49,50,51,52,53,54,55,56,57,58,59,60,61,62,63,64] (File S1 Table S1). Of these 40 articles, 14 had a last-author affiliation from Asia (35%), 16 from Europe (40%), and 10 from North America (25%). All 40 manuscripts used the term “randomization” to describe their treatment allocation in the title and/or abstract. In the full text, 16 articles were identified as using the randomization allocation procedure (9 used stratified block randomization, 7 had no stratification variable mentioned), 10 as using minimization, and the remaining 14 did not clearly report the method used for allocation.

Table 2 summarizes the characteristics of the studies included. The median number of patients was 109 (range: 24–376) with 2 arms in median (range: 2–4). Twenty-seven trials used stratification variable(s) in the allocation procedure: nine of the sixteen that used randomization, ten that used minimization, and eight out of fourteen in the unspecified method group. Overall, the median number of variables used as a stratification variable was 1 (0–3) for studies using randomization as the allocation procedure and 3 (1–7) for those using minimization.

Out of the 40 studies included, 31 were multicenter. The median numbers of centers were, respectively, 3 (1–36) and 29 (1–63) for the randomization and minimization groups. The center was a stratification variable in 10 studies (3 using randomization, 6 using minimization, and 1 using an unspecified method). In these 10 studies, the median number of centers was high (18 for randomization, 39 for minimization studies, and 9 for the unspecified-method studies).

Of the 40 studies included, 33 (82.5%) reported comparisons between arms, 14 out of the 16 that used randomization and 8 out of the 10 that used minimization. Of them, eight mentioned statistical methods for including stratification variables in their comparisons (using adjustment or subgroup comparisons on stratification variables).

Manuscripts using SPBR or minimization were published in journals with similar impact factors and the same rankings.

### 3.2. Imbalance in Real Clinical Trial Data

From the individual characteristics of each of the six real trials (14–19), we simulated the allocation arm 1000 times, using the minimization and SPBR methods, to observe an imbalance in the baseline characteristics between arms. The parameters and objectives of each study are available in the Supplemental Materials (File S2, Table S1).

**Figure 3:**
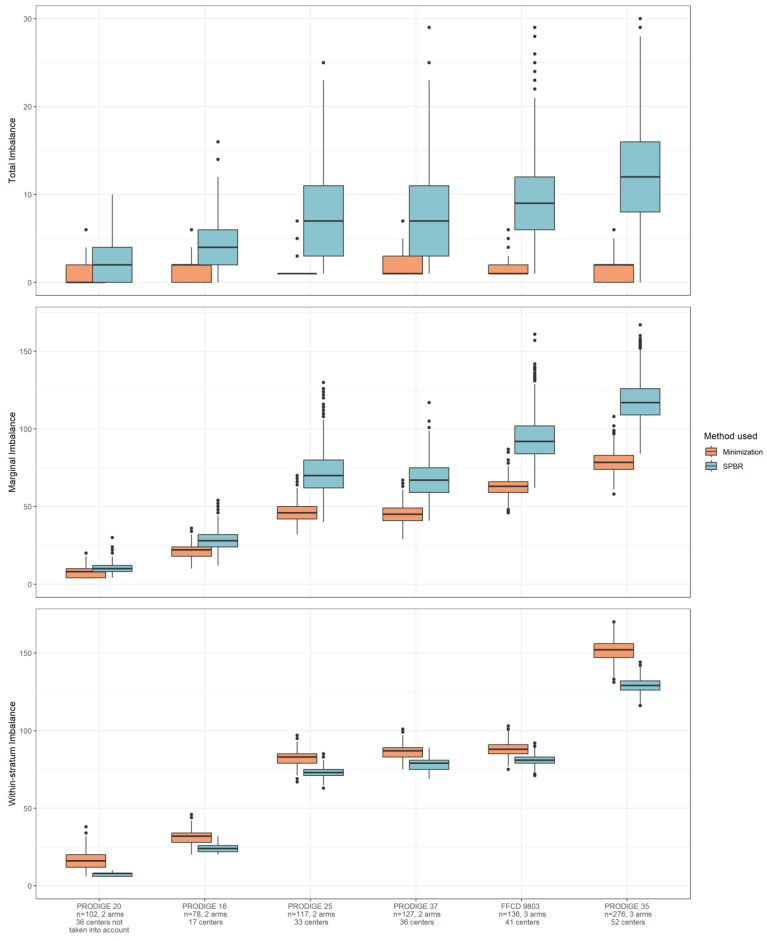
Simulation of re-randomized real databases. The impact on imbalance depending on the selected method. For each trial, the distribution of the imbalances (total, marginal, and within-stratum) calculated in the 1000 simulated data sets for the 2 allocation arm methods (minimization and SPBR) is presented: boxes correspond to the interquartile range imbalance (25th–75th percentiles), the central segment corresponds to the median imbalance, and the whiskers are the lines that extend from the top or the bottom of the box extending to 1.5 times the interquartile range, bullets correspond to outliers.

In all studies, we observed similar patterns of imbalance distribution: using SPBR gave higher total and marginal imbalances. SPBR produced a lower within-stratum imbalance than minimization did (Figure 3 and File S2, Table S2), as it is primarily based on controlling the within-stratum imbalance as much as possible.

In the studies with the highest number of centers, the marginal and within-stratum imbalances increased with both methods; in such situations, the total imbalance only increased when using SPBR. For the studies with the highest number of centers, the total and marginal imbalances in the SPBR group were higher than with minimization, while the within-stratum imbalance was higher with minimization. We noted that the boxes were increasingly separated when the number of centers increased, without overlapping.

When centers were not included as stratification variables in the simulated data sets, both imbalance values and differences in imbalance between SPBR and minimization were reduced for all three levels of imbalance (File S2, Figure S1, and Table S3).

### 3.3. Impact on Endpoint Evaluation in Real Clinical Trial Data

When we simulated the primary endpoint based on the individual data from PRODIGE 37, the treatment effects within each stratum and the efficacy results were similar between the two methods. For PRODIGE 35, the bias ranged from −1.2 to 0.8, with no method systematically obtaining a bias closer to 0. For PRODIGE 37, the bias was only positive; in each arm, the 6-month PFS rate assessed in the simulations was therefore on average lower than that published, without any change in the trial conclusion. Neither method outperformed the other in these two simulations with regard to the evaluation of the endpoint (File S2, Figure S2, and Table S4).

## 4. Discussion

### 4.1. Literature Review (Main Finding)

Randomization appears to be the best known and most popular way of ensuring that any baseline differences between groups are solely the result of randomness. This is essential for trusting the results observed in different groups. It is so ingrained in the scientific reasoning in medical clinical research that the absence of the word “randomized” in the title may be viewed with suspicion. We wonder if the absence of the word “randomized” may lead to manuscripts being rejected or grants not being awarded if reviewers are not well aware of biostatistics.

In our literature review, we showed that often, the allocation method is insufficiently described, especially when stratification variables are used. In studies with stratification, 30% did not specify the method used to implement the stratification in allocating the treatment arm. Only 1 out of 10 reported the random factor value in case of minimization. We observed that sometimes, the use of the word “minimization” is not documented in the article itself but only used in the Statistical Analysis Plan. The term “randomization” may not be appropriate for describing the allocation of arms when minimization is used without containing a random component. A moderate recommended proportion (between 10 and 25%) should be subject to randomness and described in the method sections with the help of a biostatistician. A recent simulation study [65] showed that minimization insufficiently controls serious covariate imbalances for 35% of randomness.

One limitation of this literature review is that it is restricted solely to gastrointestinal oncology studies. We cannot exclude the possibility that different results could be observed for other primary tumor sites; for instance, rare site localizations may impact the recruitment and design selection

### 4.2. Imbalance in Real Clinical Trial Data

We found that the total and marginal imbalances were greater with SPBR than with minimization, except when centers were not taken into account (File S2, Figure S1, and Table S3). Minimization decreased these imbalances but also appeared to increase the within-stratum imbalance.

Considering prognostic factors when estimating efficacy is essential in phase II trials. By ensuring that the control arm is as close as possible to the experimental arm, one must guarantee that the effects of prognostic factors other than treatment are identified. More specifically, when the number of patients per arm is different (total imbalance), the precision of the estimate (confidence interval) is influenced. When there is an imbalance between arms for a stratification covariable (marginal imbalance), the estimate in one arm may be biased by the prognostic effect of this covariable (such as the performance status, center, etc.). When there is an imbalance within a stratum, the estimate in one arm may be biased by the interaction of these two covariables between each other. In practice, we believe that in phase II clinical trials, the most important imbalance to avoid is the total imbalance and the marginal imbalance in the case of stratification. In our clinical trial data analyses, we found that minimization outperforms SPBR regarding these two points, especially when stratification by center is desired.

We encountered difficulties simulating the efficacy within each stratum from real clinical data: the number of patients in each stratum was sometimes very small (1 or 0 patients). As we treated the endpoint as a binary variable (and not as a rate from a survival distribution), the proportion of patients that reached success in some strata was sometimes 1/1 = 100%. The precision of the efficacy estimate was not sufficient to draw conclusions on the differences in efficacy estimates for each method.

### 4.3. Center as a Stratification Variable

The center is frequently considered a prognostic factor by itself. We found that 78% of the trials reported in our review were multicenter, with a median of 11 centers. In the case of multicenter trials, centers must thus be considered a stratification variable. When numerous centers are used as a stratification factor, we have shown that the marginal imbalance of arm allocation is high, and the efficacy estimation may thus be influenced by the specificity of these centers. In practice, it may be difficult to include centers in the stratification variables if SPBR is used (if there are too many, this would generate too many incomplete or empty blocks), and that will negatively affect the total imbalance of the study and thus the similarity of both arms. In situations with multiple centers, minimization has better results, with a systematically lower imbalance; we therefore consider that minimization must be recommended in the case of multicenter studies.

We found that SPBR performs better than minimization regarding within-stratum imbalance. These results warn us about a potential bias due to interactions of stratified variables on the treatment effect. Looking at treatment effects at the intersection of different categories of variables is not relevant in phase II trials, as these studies are not designed for this purpose. As phase II trials have fewer participants than phase III trials, the estimation of efficacy within a stratum is not possible. However, in larger phase III clinical trials, when there are more patients in each subgroup, the estimation of efficacy within a stratum may be possible, and we thus recommend using SPBR instead of minimization.

Recently, in a journal specializing in methodology and statistics, Coart et al. [66] reported a tutorial overviewing methodological and statistical issue according to the choice of allocation arm method, especially with minimization. Specifically, they addressed the two most common limits imputed to the minimization method: predictability and Type-I error control. In detail, using a simulation study and review of 50 trials using minimization conducted in their center, they showed that the predictability of the allocation arm (the probability of correctly guessing the next treatment allocation) is only an issue in the uncommon scenario where investigators participating in the trial have knowledge of the treatment allocation algorithm and information according to treatment group on the patients allocated so far. In this specific situation, modified algorithms are available to reduce predictability. Callegaro et al. [67] showed that the randomization test preserves type-I errors, and Coart et al. showed that the randomization test and usual asymptotic tests provide similar inferences. Finally, they concluded that minimization is especially useful when many baseline factors are known to have an impact on the patient prognosis and in small or medium sample sizes (up to 100 patients), which is a common situation when planning phase II studies.

### 4.4. Other Methods

In this report, our primary focus is on SPBR and minimization; however, there are various approaches employed in clinical trial designs. Among these, “pick-the-winner” is a response-adaptive design that adjusts treatment assignment probabilities based on observed outcomes during the trial, with the aim of allocating more participants to treatments demonstrating superior efficacy. Biomarker-Adaptive Randomization tailors treatment allocation according to participants’ biomarker profiles, potentially enhancing treatment efficacy by targeting specific biological characteristics. Bayesian Adaptive Randomization utilizes Bayesian statistical methods to update treatment allocation probabilities, integrating prior knowledge and offering a flexible framework for adjusting randomization based on accumulating trial data. Each method presents distinct advantages and considerations, contributing to the comprehensive toolkit that is available for optimizing clinical trial designs and execution. It would be of interest in future studies to explore these alternative methods of arm allocation further.

## 5. Conclusions

The minimization method offers a better opportunity to guarantee similarity between treatment arms, while Stratified Permuted Block Randomization may be less effective, particularly in terms of the total and marginal imbalances. This is particularly true in cases of multicenter trials and in phase 2 clinical trials with a relatively small sample size. As phase II trials do not focus on demonstrating the superiority of one arm, or on the effects of the intersection of variables or modeling efficacy, the total and marginal imbalances should be minimized as a priority. This method is easily understandable and directly applicable to clinical studies, as it is implemented in most statistical or clinical database management software programs.

## Figures and Tables

**Table 1:** Example of distribution of patients (*n* = 100) using 2 stratification variables (center, smoking). The 3 types of imbalances are shown in gray.

Stratification Variable 1	Stratification Variable 2	ARM A	ARM B	Difference
Total		50	50	0 (Total imbalance)
Total Center 1		15	15	0
Total Center 2		11	12	1
Total Center 3		24	23	1
Total Non-smoker		19	19	0
Total Smoker		31	31	0
				2 (Marginal imbalance)
Center 1	Non-smoker	7	3	4
	Smoker	8	12	4
Center 2	Non-smoker	3	6	3
	Smoker	8	6	2
Center 3	Non-smoker	9	10	1
	Smoker	15	13	2
		Subtotal		16 (Within-stratum imbalance)

**Table 2:** Description of studies in the literature review.

	All Studies	According to Treatment Allocation Procedure			Studies with at Least One Stratification Variable *n* = 27			Studies Using Center as Stratification Variable *n* = 10		
	All	Randomization	Minimization	Unspecified	Randomization	Minimization	Unspecified	Randomization	Minimization	Unspecified
	*n* = 40	*n* = 16	*n* = 10	*n* = 14	*n* = 9	*n* = 10	*n* = 8	*n* = 3	*n* = 6	*n* = 1
Number of patients randomized										
Median (min–max)	109 (24–376)	122 (29–311)	117.5 (67–280)	72.5 (24–376)	152 (82.0–311.0)	117.5 (67–280)	105.5 (24–376)	160 (82–311)	128 (101–280)	81
Number of arms										
Median (min–max)	2 (2–4)	2 (2–4)	2 (2–4)	2 (2–2)	2 (2–4)	2 (2–4)	2 (2–2)	2 (2–2)	2.5 (2–4)	2
Number of stratification variables										
Median (min–max)	2 (0–7) *¥*	1 (0–3) *¥*	3 (1–7)	2 (0–4)	2 (1–3) *¥*	3 (1–7)	2.5 (2–4)	2 (1–2)	3 (1–4)	3
Number of centers										
Median (min–max)	11 (1–63)	3 (1–36)	29 (1–63) *§*	10 (1–60)	3 (1–36)	29 (1–63) *§*	22.5 (1–60)	18 (3–23)	39 (25–53) *¥*	9
>1 center	31 (77.5%)	11 (68.8%)	9 (90%)	11 (78.6%)	6 (75%)	9 (90%)	7 (87.5%)			
Center as a stratification variable										
Yes	10 (25.6%)	3 (20%) *¥*	6 (60%)	1 (7.1%)	3 (37.5%) *¥*	6 (60%)	1 (12.5%)			
Other geographical unit as stratification variable										
Yes	5 (12.8%)	1 (6.7%) *¥*	0 (0%)	4 (28.6%)	1 (12.5%) *¥*	0 (0%)	4 (50%)			
Statistical comparison between arms										
Yes	33 (82.5%)	14 (87.5%)	8 (80%)	11 (78.6%)	6 (75%) 7 (77.8%)	8 (80%)	7 (87.5%)	2 (66.7%)	5 (83.3%)	1
Adjustments, stratified or subgroup analyses using stratification variable										
Yes	8 (20.5%) *¥*	3 (20%) *¥*	2 (20%)	3 (21.4%)	2 (25%) *¥*	2 (20%)	3 (37.5%)	1 (33.3%)	2 (33.3%)	0

*¥*: One study did not report the details of the stratification variables or their number (id 41); *§*: one study indicated a multicenter trial but did not report the exact number of centers (id 20).

## Data Availability

The data sets used and/or analyzed during the current study are available from the FFCD and PRODIGE intergroup on reasonable request.

## Supplementary Materials

The following supporting information can be downloaded at https://www.mdpi.com/article/10.3390/curroncol31060259/s1, File S1. Contains information about the literature review (DOCX). File S2. Contains information about simulations using real data studies (DOCX). References [25,26,27,28,29,30,31,32,33,34,35,36,37,38,39,40,41,42,43,44,45,46,47,48,49,50,51,52,53,54,55,56,57,58,59,60,61,62,63,64] are cited in the Supplementary Materials.

## References

[1] Rubinstein L.V., Korn E.L., Freidlin B., Hunsberger S., Ivy S.P., Smith M.A. (2005). Design Issues of Randomized Phase II Trials and a Proposal for Phase II Screening Trials. J. Clin. Oncol..

[2] (1997). Phase II Trials in the EORTC. The Protocol Review Committee, the Data Center, the Research and Treatment Division, and the New Drug Development Office. European Organization for Research and Treatment of Cancer. Eur. J. Cancer.

[3] (1999). ICH Harmonised Tripartite Guideline. Statistical Principles for Clinical Trials. International Conference on Harmonisation E9 Expert Working Group. Stat. Med..

[4] Guyatt G.H., Alexander P.E. (2013). Randomization. Important Considerations for Clinical Trial Methodologies.

[5] Chia K.S. (2000). Randomisation: Magical Cure for Bias?. Ann. Acad. Med. Singap..

[6] Sedgwick P. (2015). Treatment Allocation in Trials: Stratified Randomisation. BMJ.

[7] Matts J.P., Lachin J.M. (1988). Properties of Permuted-Block Randomization in Clinical Trials. Control Clin. Trials.

[8] Treasure T., MacRae K.D. (1998). Minimisation: The Platinum Standard for Trials?: Randomisation Doesn’t Guarantee Similarity of Groups; Minimisation Does. BMJ.

[9] Taves D.R. (1974). Minimization: A New Method of Assigning Patients to Treatment and Control Groups. Clin. Pharmacol. Ther..

[10] Pocock S.J., Simon R. (1975). Sequential Treatment Assignment with Balancing for Prognostic Factors in the Controlled Clinical Trial. Biometrics.

[11] Altman D.G., Schulz K.F., Moher D., Egger M., Davidoff F., Elbourne D., Gøtzsche P.C., Lang T., CONSORT GROUP (Consolidated Standards of Reporting Trials) (2001). The Revised CONSORT Statement for Reporting Randomized Trials: Explanation and Elaboration. Ann. Intern. Med..

[12] Altman D.G. (1990). Practical Statistics for Medical Research.

[13] Altman D.G., Bland J.M. (2005). Treatment Allocation by Minimisation. BMJ.

[14] Brown S., Thorpe H., Hawkins K., Brown J. (2005). Minimization—Reducing Predictability for Multi-Centre Trials Whilst Retaining Balance within Centre. Stat. Med..

[15] Tu F., Ye X., Ma W., Hu F. (2023). Carat: Covariate-Adaptive Randomization for Clinical Trials. J. Stat. Softw..

[16] European Medicines Agency (2015). Guideline on Adjustment for Baseline Covariates in Clinical Trials.

[17] Bouché O., Raoul J.L., Bonnetain F., Giovannini M., Etienne P.L., Lledo G., Arsène D., Paitel J.F., Guérin-Meyer V., Mitry E. (2004). Randomized Multicenter Phase II Trial of a Biweekly Regimen of Fluorouracil and Leucovorin (LV5FU2), LV5FU2 Plus Cisplatin, or LV5FU2 Plus Irinotecan in Patients with Previously Untreated Metastatic Gastric Cancer: A Fédération Francophone de Cancérologie Digestive Group Study—FFCD 9803. JCO.

[18] Turpin A., de Baere T., Heurgué A., Le Malicot K., Ollivier-Hourmand I., Lecomte T., Perrier H., Vergniol J., Sefrioui D., Rinaldi Y. (2021). Liver Transarterial Chemoembolization and Sunitinib for Unresectable Hepatocellular Carcinoma: Results of the PRODIGE 16 Study. Clin. Res. Hepatol. Gastroenterol..

[19] Aparicio T., Bouché O., Taieb J., Maillard E., Kirscher S., Etienne P.-L., Faroux R., Khemissa Akouz F., El Hajbi F., Locher C. (2018). Bevacizumab+chemotherapy versus Chemotherapy Alone in Elderly Patients with Untreated Metastatic Colorectal Cancer: A Randomized Phase II Trial—PRODIGE 20 Study Results. Ann. Oncol..

[20] Legoux J.L., Faroux R., Barriere N., Le Malicot K., Tougeron D., Lorgis V., Guérin-Meyer V., Bourgeois V., Malka D., Aparicio T. (2020). 444P PRODIGE 25 (FFCD 11-01)-FOLFA: A Randomized Phase II Trial Evaluating Aflibercept Associated with LV5FU2 Regimen as First-Line Treatment of Non-Resectable Metastatic Colorectal Cancers. Ann. Oncol..

[21] Dahan L., Williet N., Le Malicot K., Phelip J.-M., Desrame J., Bouché O., Petorin C., Malka D., Rebischung C., Aparicio T. (2021). Randomized Phase II Trial Evaluating Two Sequential Treatments in First Line of Metastatic Pancreatic Cancer: Results of the PANOPTIMOX-PRODIGE 35 Trial. J. Clin. Oncol..

[22] Rinaldi Y., Pointet A.L., Khemissa Akouz F., Le Malicot K., Wahiba B., Louafi S., Gratet A., Miglianico L., Laharie H., Bouhier Lepoirrier K. (2020). Gemcitabine plus Nab-Paclitaxel until Progression or Alternating with FOLFIRI.3, as First-Line Treatment for Patients with Metastatic Pancreatic Adenocarcinoma: The Federation Francophone de CancErologie Digestive-PRODIGE 37 Randomised Phase II Study (FIRGEMAX). Eur. J. Cancer.

[23] Jin M., Polis A., Hartzel J. (2021). Algorithms for minimization randomization and the implementation with a R package. Commun. Stat. Simul. Comput..

[24] Snow G. (2022). Blockrand: Randomization for Block Random Clinical Trials. version 1.5. https://cran.r-project.org/web/packages/blockrand/index.html.

[25] Ng K., Nimeiri H.S., McCleary N.J., Abrams T.A., Yurgelun M.B., Cleary J.M., Rubinson D.A., Schrag D., Miksad R., Bullock A.J. (2019). Effect of High-Dose vs Standard-Dose Vitamin D3 Supplementation on Progression-Free Survival Among Patients With Advanced or Metastatic Colorectal Cancer: The SUNSHINE Randomized Clinical Trial. JAMA.

[26] Wang X.S., Shi Q., Bhadkamkar N.A., Cleeland C.S., Garcia-Gonzalez A., Aguilar J.R., Heijnen C., Eng C. (2019). Minocycline for Symptom Reduction During Oxaliplatin-Based Chemotherapy for Colorectal Cancer: A Phase II Randomized Clinical Trial. J. Pain Symptom Manag..

[27] Bjerring O.S., Fristrup C.W., Pfeiffer P., Lundell L., Mortensen M.B. (2019). Phase II Randomized Clinical Trial of Endosonography and PET/CT versus Clinical Assessment Only for Follow-up after Surgery for Upper Gastrointestinal Cancer (EUFURO Study). Br. J. Surg..

[28] Bednarski B.K., Nickerson T.P., You Y.N., Messick C.A., Speer B., Gottumukkala V., Manandhar M., Weldon M., Dean E.M., Qiao W. (2019). Randomized Clinical Trial of Accelerated Enhanced Recovery after Minimally Invasive Colorectal Cancer Surgery (RecoverMI Trial). Br. J. Surg..

[29] Tanaka Y., Yamada A., Hirata S., Tanaka H., Sakuratani T., Matsuhashi N., Yamaguchi K., Shimokawa T., Yoshida K. (2019). Efficacy and Safety of Enoxaparin for Prophylaxis of Postoperative Venous Thromboembolism After Esophagectomy: A Single-Center Prospective Randomized Controlled Phase II Study. Anticancer Res..

[30] Taghizadeh Kermani A., Hosseini S., Fanipakdel A., Joudi Mashhad M., Akhavan Rezayat K., Zardadi M., Gholami A., Javadinia S.A., Ferns G.A., Avan A. (2019). A Randomized Clinical Trial on the Antitumoral Effects of Low Molecular Weight Heparin in the Treatment of Esophageal Cancer. J. Cell. Physiol..

[31] Curtis N.J., Conti J.A., Dalton R., Rockall T.A., Allison A.S., Ockrim J.B., Jourdan I.C., Torkington J., Phillips S., Allison J. (2019). 2D versus 3D Laparoscopic Total Mesorectal Excision: A Developmental Multicentre Randomised Controlled Trial. Surg. Endosc..

[32] Schmelz R., Miehlke S., Thiede C., Brueckner S., Dawel M., Kuhn M., Ruskoné-Formestraux A., Stolte M., Jentsch C., Hampe J. (2019). Sequential H. Pylori Eradication and Radiation Therapy with Reduced Dose Compared to Standard Dose for Gastric MALT Lymphoma Stages IE & II1E: A Prospective Randomized Trial. J. Gastroenterol..

[33] Boku N., Ryu M.-H., Kato K., Chung H.C., Minashi K., Lee K.-W., Cho H., Kang W.K., Komatsu Y., Tsuda M. (2019). Safety and Efficacy of Nivolumab in Combination with S-1/Capecitabine plus Oxaliplatin in Patients with Previously Untreated, Unresectable, Advanced, or Recurrent Gastric/Gastroesophageal Junction Cancer: Interim Results of a Randomized, Phase II Trial (ATTRACTION-4). Ann. Oncol..

[34] Howells L.M., Iwuji C.O.O., Irving G.R.B., Barber S., Walter H., Sidat Z., Griffin-Teall N., Singh R., Foreman N., Patel S.R. (2019). Curcumin Combined with FOLFOX Chemotherapy Is Safe and Tolerable in Patients with Metastatic Colorectal Cancer in a Randomized Phase IIa Trial. J. Nutr..

[35] Ghiringhelli F., Vincent J., Bengrine L., Borg C., Jouve J.L., Loffroy R., Guiu B., Blanc J., Bertaut A. (2019). Hepatic Arterial Chemotherapy with Raltitrexed and Oxaliplatin versus Standard Chemotherapy in Unresectable Liver Metastases from Colorectal Cancer after Conventional Chemotherapy Failure (HEARTO): A Randomized Phase-II Study. J. Cancer Res. Clin. Oncol..

[36] Cremolini C., Marmorino F., Bergamo F., Aprile G., Salvatore L., Masi G., Dell’Aquila E., Antoniotti C., Murgioni S., Allegrini G. (2019). Phase II Randomised Study of Maintenance Treatment with Bevacizumab or Bevacizumab plus Metronomic Chemotherapy after First-Line Induction with FOLFOXIRI plus Bevacizumab for Metastatic Colorectal Cancer Patients: The MOMA Trial. Eur. J. Cancer.

[37] Wang J., Guan Y., Gu W., Yan S., Zhou J., Huang D., Tong T., Li C., Cai S., Zhang Z. (2019). Long-Course Neoadjuvant Chemoradiotherapy with versus without a Concomitant Boost in Locally Advanced Rectal Cancer: A Randomized, Multicenter, Phase II Trial (FDRT-002). Radiat. Oncol..

[38] Yu P., Du Y., Xu Z., Huang L., Cheng X. (2019). Comparison of D2 and D2 plus Radical Surgery for Advanced Distal Gastric Cancer: A Randomized Controlled Study. World J. Surg. Oncol..

[39] Bekaii-Saab T.S., Ou F.-S., Ahn D.H., Boland P.M., Ciombor K.K., Heying E.N., Dockter T.J., Jacobs N.L., Pasche B.C., Cleary J.M. (2019). Regorafenib Dose-Optimisation in Patients with Refractory Metastatic Colorectal Cancer (ReDOS): A Randomised, Multicentre, Open-Label, Phase 2 Study. Lancet Oncol..

[40] Bennouna J., Hiret S., Bertaut A., Bouché O., Deplanque G., Borel C., François E., Conroy T., Ghiringhelli F., des Guetz G. (2019). Continuation of Bevacizumab vs Cetuximab Plus Chemotherapy After First Progression in KRAS Wild-Type Metastatic Colorectal Cancer: The UNICANCER PRODIGE18 Randomized Clinical Trial. JAMA Oncol..

[41] Shitara K., Yamanaka T., Denda T., Tsuji Y., Shinozaki K., Komatsu Y., Kobayashi Y., Furuse J., Okuda H., Asayama M. (2019). REVERCE: A Randomized Phase II Study of Regorafenib Followed by Cetuximab versus the Reverse Sequence for Previously Treated Metastatic Colorectal Cancer Patients. Ann. Oncol..

[42] Pietrantonio F., Lobefaro R., Antista M., Lonardi S., Raimondi A., Morano F., Mosconi S., Rimassa L., Murgioni S., Sartore-Bianchi A. (2020). Capecitabine and Temozolomide versus FOLFIRI in RAS-Mutated, MGMT-Methylated Metastatic Colorectal Cancer. Clin. Cancer Res. Off. J. Am. Assoc. Cancer Res..

[43] Oki E., Emi Y., Yamanaka T., Uetake H., Muro K., Takahashi T., Nagasaka T., Hatano E., Ojima H., Manaka D. (2019). Randomised Phase II Trial of MFOLFOX6 plus Bevacizumab versus MFOLFOX6 plus Cetuximab as First-Line Treatment for Colorectal Liver Metastasis (ATOM Trial). Br. J. Cancer.

[44] Malka D., François E., Penault-Llorca F., Castan F., Bouché O., Bennouna J., Ghiringhelli F., de la Fouchardière C., Borg C., Samalin E. (2019). FOLFOX Alone or Combined with Rilotumumab or Panitumumab as First-Line Treatment for Patients with Advanced Gastroesophageal Adenocarcinoma (PRODIGE 17-ACCORD 20-MEGA): A Randomised, Open-Label, Three-Arm Phase II Trial. Eur. J. Cancer.

[45] Adenis A., Bennouna J., Etienne P.L., Bogart E., Francois E., Galais M.P., Ben Abdelghani M., Michel P., Metges J.P., Dahan L. (2019). Continuation versus Discontinuation of First-Line Chemotherapy in Patients with Metastatic Squamous Cell Oesophageal Cancer: A Randomised Phase II Trial (E-DIS). Eur. J. Cancer.

[46] Munemoto Y., Nakamura M., Takahashi M., Kotaka M., Kuroda H., Kato T., Minagawa N., Noura S., Fukunaga M., Kuramochi H. (2019). SAPPHIRE: A Randomised Phase II Study of Planned Discontinuation or Continuous Treatment of Oxaliplatin after Six Cycles of Modified FOLFOX6 plus Panitumumab in Patients with Colorectal Cancer. Eur. J. Cancer.

[47] Kobayashi H., Uetake H., Yasuno M., Sugihara K. (2019). Effectiveness of Wound-Edge Protectors for Preventing Surgical Site Infections after Open Surgery for Colorectal Disease: A Prospective Cohort Study with Two Parallel Study Groups. Dig. Surg..

[48] Hurwitz H.I., Tan B.R., Reeves J.A., Xiong H., Somer B., Lenz H.-J., Hochster H.S., Scappaticci F., Palma J.F., Price R. (2019). Phase II Randomized Trial of Sequential or Concurrent FOLFOXIRI-Bevacizumab Versus FOLFOX-Bevacizumab for Metastatic Colorectal Cancer (STEAM). Oncologist.

[49] Fokas E., Allgäuer M., Polat B., Klautke G., Grabenbauer G.G., Fietkau R., Kuhnt T., Staib L., Brunner T., Grosu A.-L. (2019). Randomized Phase II Trial of Chemoradiotherapy Plus Induction or Consolidation Chemotherapy as Total Neoadjuvant Therapy for Locally Advanced Rectal Cancer: CAO/ARO/AIO-12. J. Clin. Oncol..

[50] Modest D.P., Martens U.M., Riera-Knorrenschild J., Greeve J., Florschütz A., Wessendorf S., Ettrich T., Kanzler S., Nörenberg D., Ricke J. (2019). FOLFOXIRI Plus Panitumumab As First-Line Treatment of RAS Wild-Type Metastatic Colorectal Cancer: The Randomized, Open-Label, Phase II VOLFI Study (AIO KRK0109). J. Clin. Oncol..

[51] Kim C., Chon H.J., Kim J.H., Jung M., Nam C.M., Kim H.S., Kang B., Chung H.C., Rha S.Y. (2019). Randomised Phase II Trial Comparing Four Front-Line Doublets in Asian Patients with Metastatic Gastric Cancer. Eur. J. Cancer.

[52] Yoshikawa T., Muro K., Shitara K., Oh D.-Y., Kang Y.-K., Chung H.C., Kudo T., Chin K., Kadowaki S., Hamamoto Y. (2019). Effect of First-Line S-1 Plus Oxaliplatin with or Without Ramucirumab Followed by Paclitaxel Plus Ramucirumab on Advanced Gastric Cancer in East Asia: The Phase 2 RAINSTORM Randomized Clinical Trial. JAMA Netw. Open.

[53] Páez D., Tobeña M., Fernández-Plana J., Sebio A., Virgili A.C., Cirera L., Barnadas A., Riera P., Sullivan I., Salazar J. (2019). Pharmacogenetic Clinical Randomised Phase II Trial to Evaluate the Efficacy and Safety of FOLFIRI with High-Dose Irinotecan (HD-FOLFIRI) in Metastatic Colorectal Cancer Patients According to Their UGT1A 1 Genotype. Br. J. Cancer.

[54] McGregor L.M., Skrobanski H., Ritchie M., Berkman L., Miller H., Freeman M., Patel N., Morris S., Rees C., von Wagner C. (2019). Using Specialist Screening Practitioners (SSPs) to Increase Uptake of Bowel Scope (Flexible Sigmoidoscopy) Screening: Results of a Feasibility Single-Stage Phase II Randomised Trial. BMJ Open.

[55] Winther S.B., Liposits G., Skuladottir H., Hofsli E., Shah C.-H., Poulsen L.Ø., Ryg J., Osterlund P., Berglund Å., Qvortrup C. (2019). Reduced-Dose Combination Chemotherapy (S-1 plus Oxaliplatin) versus Full-Dose Monotherapy (S-1) in Older Vulnerable Patients with Metastatic Colorectal Cancer (NORDIC9): A Randomised, Open-Label Phase 2 Trial. Lancet Gastroenterol. Hepatol..

[56] Hamada K., Uedo N., Tonai Y., Arao M., Suzuki S., Iwatsubo T., Kato M., Shichijo S., Yamasaki Y., Matsuura N. (2019). Efficacy of Vonoprazan in Prevention of Bleeding from Endoscopic Submucosal Dissection-Induced Gastric Ulcers: A Prospective Randomized Phase II Study. J. Gastroenterol..

[57] Kienle D.L., Dietrich D., Ribi K., Wicki A., Quagliata L., Winterhalder R.C., Koeberle D., Horber D., Bastian S., Kueng M. (2019). Cetuximab Monotherapy and Cetuximab plus Capecitabine as First-Line Treatment in Older Patients with RAS- and BRAF Wild-Type Metastatic Colorectal Cancer. Results of the Multicenter Phase II Trial SAKK 41/10. J. Geriatr. Oncol..

[58] Parikh A.R., Lee F.-C., Yau L., Koh H., Knost J., Mitchell E.P., Bosanac I., Choong N., Scappaticci F., Mancao C. (2019). MAVERICC, a Randomized, Biomarker-Stratified, Phase II Study of MFOLFOX6-Bevacizumab versus FOLFIRI-Bevacizumab as First-Line Chemotherapy in Metastatic Colorectal Cancer. Clin. Cancer Res..

[59] Yamazaki K., Ariyoshi N., Miyauchi H., Ohira G., Kaneya N., Yamamoto K., Arai K., Yamazaki S., Matsubara H., Suzuki T. (2019). A Randomized Controlled, Open-Label Early Phase II Trial Comparing Incidence of FOLFIRI.3-Induced Diarrhoea between Hangeshashinto and Oral Alkalization in Japanese Patients with Colorectal Cancer. J. Clin. Pharm. Ther..

[60] Suwa Y., Watanabe J., Ota M., Suzuki S., Suwa H., Watanabe K., Saito S., Nagamine K., Momiyama M., Ishibe A. (2019). Randomized Phase II Trial of the Prophylactic Use of Celecoxib for the Prevention of Oxaliplatin-Related Peripheral Vascular Pain in Capeox (YCOG1205). Cancer Chemother. Pharmacol..

[61] Bang Y.-J., Kang Y.-K., Ng M., Chung H.C., Wainberg Z.A., Gendreau S., Chan W.Y., Xu N., Maslyar D., Meng R. (2019). A Phase II, Randomised Study of MFOLFOX6 with or without the Akt Inhibitor Ipatasertib in Patients with Locally Advanced or Metastatic Gastric or Gastroesophageal Junction Cancer. Eur. J. Cancer.

[62] Cleary J.M., Horick N.K., McCleary N.J., Abrams T.A., Yurgelun M.B., Azzoli C.G., Rubinson D.A., Brooks G.A., Chan J.A., Blaszkowsky L.S. (2019). FOLFOX plus Ziv-Aflibercept or Placebo in First-Line Metastatic Esophagogastric Adenocarcinoma: A Double-Blind, Randomized, Multicenter Phase 2 Trial. Cancer.

[63] Gorbunova V., Beck J.T., Hofheinz R.-D., Garcia-Alfonso P., Nechaeva M., Cubillo Gracian A., Mangel L., Elez Fernandez E., Deming D.A., Ramanathan R.K. (2019). A Phase 2 Randomised Study of Veliparib plus FOLFIRI±bevacizumab versus Placebo plus FOLFIRI±bevacizumab in Metastatic Colorectal Cancer. Br. J. Cancer.

[64] Bendell J.C., Sauri T., Gracián A.C., Alvarez R., López-López C., García-Alfonso P., Hussein M., Miron M.-L.L., Cervantes A., Montagut C. (2020). The McCAVE Trial: Vanucizumab plus MFOLFOX-6 Versus Bevacizumab plus MFOLFOX-6 in Patients with Previously Untreated Metastatic Colorectal Carcinoma (MCRC). Oncologist.

[65] Lauzon S.D., Zhao W., Nietert P.J., Ciolino J.D., Hill M.D., Ramakrishnan V. (2022). Impact of Minimal Sufficient Balance, Minimization, and Stratified Permuted Blocks on Bias and Power in the Estimation of Treatment Effect in Sequential Clinical Trials with a Binary Endpoint. Stat. Methods Med. Res..

[66] Coart E., Bamps P., Quinaux E., Sturbois G., Saad E.D., Burzykowski T., Buyse M. (2023). Minimization in Randomized Clinical Trials. Stat. Med..

[67] Callegaro A., Harsha Shree B.S., Karkada N. (2021). Inference under Covariate-Adaptive Randomization: A Simulation Study. Stat. Methods Med. Res..

