# Supplementary material for: How to Balance Prognostic Factors in Controlled Phase II Trials: Stratified Permuted Block Randomization or Minimization? An Analysis of Clinical Trials in Digestive Oncology
Source: Curr Oncol. 2024 Jun 17;31(6):3513–28. doi: 10.3390/curroncol31060259 (PMC11202503; doi:10.3390/curroncol31060259)
Supplement: Supplementary file 1 [file curroncol-31-00259-s001.zip › curroncol-3034724-supplementary/File S1.pdf]

Supplementary material S1

List S1 : Search algorithm on Medline (PUBMED)

(((("english"[Language]) AND ("clinical trial, phase ii"[Publication Type])) AND (gastrointestinal neoplasms [MeSH Terms])) AND (("2019/01/01"[Date - Publication] : "2019/12/01"[Date - Publication]))) AND ("controlled clinical trial"[Publication Type]).

Table S1: References of selected studies in literature review

	Minimization	Randomization	Unspecified
**Studies without stratification variable used for allocation arm**			
	-	[25–32]	[33–38]
**Studies using stratification variable for allocation arm**			
	[39–48]	Taghizadeh Kermani et al. 2019; [49–56]	[57–64]

25. Ng, K.; Nimeiri, H.S.; McCleary, N.J.; Abrams, T.A.; Yurgelun, M.B.; Cleary, J.M.; Rubinson, D.A.; Schrag, D.; Miksad, R.; Bullock, A.J.; et al. Effect of High-Dose vs Standard-Dose Vitamin D3 Supplementation on Progression-Free Survival Among Patients With Advanced or Metastatic Colorectal Cancer: The SUNSHINE Randomized Clinical Trial. JAMA 2019, 321, 1370–1379, doi:10.1001/jama.2019.2402.
26. Wang, X.S.; Shi, Q.; Bhadkamkar, N.A.; Cleeland, C.S.; Garcia-Gonzalez, A.; Aguilar, J.R.; Heijnen, C.; Eng, C. Minocycline for Symptom Reduction During Oxaliplatin-Based Chemotherapy for Colorectal Cancer: A Phase II Randomized Clinical Trial. J. Pain Symptom Manage. 2019, 58, 662–671, doi:10.1016/j.jpainsymman.2019.06.018.
27. Bjerring, O.S.; Fristrup, C.W.; Pfeiffer, P.; Lundell, L.; Mortensen, M.B. Phase II Randomized Clinical Trial of Endosonography and PET/CT versus Clinical Assessment Only for Follow-up after Surgery for Upper Gastrointestinal Cancer (EUFURO Study). Br. J. Surg. 2019, 106, 1761–1768, doi:10.1002/bjs.11290.
28. Bednarski, B.K.; Nickerson, T.P.; You, Y.N.; Messick, C.A.; Speer, B.; Gottumukkala, V.; Manandhar, M.; Weldon, M.; Dean, E.M.; Qiao, W.; et al. Randomized Clinical Trial of Accelerated Enhanced Recovery after Minimally Invasive Colorectal Cancer Surgery (RecoverMI Trial). Br. J. Surg. 2019, 106, 1311–1318, doi:10.1002/bjs.11223.
29. Tanaka, Y.; Yamada, A.; Hirata, S.; Tanaka, H.; Sakuratani, T.; Matsuhashi, N.; Yamaguchi, K.; Shimokawa, T.; Yoshida, K. Efficacy and Safety of Enoxaparin for Prophylaxis of Postoperative Venous Thromboembolism After Esophagectomy: A Single-Center Prospective Randomized Controlled Phase II Study. Anticancer Res. 2019, 39, 2615–2625, doi:10.21873/anticanres.13385.
30. Taghizadeh Kermani, A.; Hosseini, S.; Fanipakdel, A.; Joudi Mashhad, M.; Akhavan Rezayat, K.; Zardadi, M.; Gholami, A.; Javadinia, S.A.; Ferns, G.A.; Avan, A. A Randomized Clinical Trial on the Antitumoral Effects of Low Molecular Weight Heparin in the

Treatment of Esophageal Cancer. J. Cell. Physiol. 2019, 234, 4191–4199, doi:10.1002/jcp.27177.
31. Curtis, N.J.; Conti, J.A.; Dalton, R.; Rockall, T.A.; Allison, A.S.; Ockrim, J.B.; Jourdan, I.C.; Torkington, J.; Phillips, S.; Allison, J.; et al. 2D versus 3D Laparoscopic Total Mesorectal Excision: A Developmental Multicentre Randomised Controlled Trial. Surg. Endosc. 2019, 33, 3370–3383, doi:10.1007/s00464-018-06630-9.
32. Schmelz, R.; Miehlke, S.; Thiede, C.; Brueckner, S.; Dawel, M.; Kuhn, M.; Ruskoné-Formestraux, A.; Stolte, M.; Jentsch, C.; Hampe, J.; et al. Sequential H. Pylori Eradication and Radiation Therapy with Reduced Dose Compared to Standard Dose for Gastric MALT Lymphoma Stages IE & II1E: A Prospective Randomized Trial. J. Gastroenterol. 2019, 54, 388–395, doi:10.1007/s00535-018-1517-4.
33. Boku, N.; Ryu, M.-H.; Kato, K.; Chung, H.C.; Minashi, K.; Lee, K.-W.; Cho, H.; Kang, W.K.; Komatsu, Y.; Tsuda, M.; et al. Safety and Efficacy of Nivolumab in Combination with S-1/Capecitabine plus Oxaliplatin in Patients with Previously Untreated, Unresectable, Advanced, or Recurrent Gastric/Gastroesophageal Junction Cancer: Interim Results of a Randomized, Phase II Trial (ATTRACTION-4). Ann. Oncol. Off. J. Eur. Soc. Med. Oncol. 2019, 30, 250–258, doi:10.1093/annonc/mdy540.
34. Howells, L.M.; Iwuji, C.O.O.; Irving, G.R.B.; Barber, S.; Walter, H.; Sidat, Z.; Griffin-Teall, N.; Singh, R.; Foreman, N.; Patel, S.R.; et al. Curcumin Combined with FOLFOX Chemotherapy Is Safe and Tolerable in Patients with Metastatic Colorectal Cancer in a Randomized Phase IIa Trial. J. Nutr. 2019, 149, 1133–1139, doi:10.1093/jn/nxz029.
35. Ghiringhelli, F.; Vincent, J.; Bengrine, L.; Borg, C.; Jouve, J.L.; Loffroy, R.; Guiu, B.; Blanc, J.; Bertaut, A. Hepatic Arterial Chemotherapy with Raltitrexed and Oxaliplatin versus Standard Chemotherapy in Unresectable Liver Metastases from Colorectal Cancer after Conventional Chemotherapy Failure (HEARTO): A Randomized Phase-II Study. J. Cancer Res. Clin. Oncol. 2019, 145, 2357–2363, doi:10.1007/s00432-019-02970-8.
36. Cremolini, C.; Marmorino, F.; Bergamo, F.; Aprile, G.; Salvatore, L.; Masi, G.; Dell'Aquila, E.; Antoniotti, C.; Murgioni, S.; Allegrini, G.; et al. Phase II Randomised Study of Maintenance Treatment with Bevacizumab or Bevacizumab plus Metronomic Chemotherapy after First-Line Induction with FOLFOXIRI plus Bevacizumab for Metastatic Colorectal Cancer Patients: The MOMA Trial. Eur. J. Cancer Oxf. Engl. 1990 2019, 109, 175–182, doi:10.1016/j.ejca.2018.12.028.
37. Wang, J.; Guan, Y.; Gu, W.; Yan, S.; Zhou, J.; Huang, D.; Tong, T.; Li, C.; Cai, S.; Zhang, Z.; et al. Long-Course Neoadjuvant Chemoradiotherapy with versus without a Concomitant Boost in Locally Advanced Rectal Cancer: A Randomized, Multicenter, Phase II Trial (FDRT-002). Radiat. Oncol. Lond. Engl. 2019, 14, 215, doi:10.1186/s13014-019-1420-z.
38. Yu, P.; Du, Y.; Xu, Z.; Huang, L.; Cheng, X. Comparison of D2 and D2 plus Radical Surgery for Advanced Distal Gastric Cancer: A Randomized Controlled Study. World J. Surg. Oncol. 2019, 17, 28, doi:10.1186/s12957-019-1572-1.
39. Bekaii-Saab, T.S.; Ou, F.-S.; Ahn, D.H.; Boland, P.M.; Ciombor, K.K.; Heying, E.N.; Dockter, T.J.; Jacobs, N.L.; Pasche, B.C.; Cleary, J.M.; et al. Regorafenib Dose-Optimisation in Patients with Refractory Metastatic Colorectal Cancer (ReDOS): A Randomised, Multicentre, Open-Label, Phase 2 Study. Lancet Oncol. 2019, 20, 1070–1082, doi:10.1016/S1470-2045(19)30272-4.

40. Bennouna, J.; Hiret, S.; Bertaut, A.; Bouché, O.; Deplanque, G.; Borel, C.; François, E.; Conroy, T.; Ghiringhelli, F.; des Guetz, G.; et al. Continuation of Bevacizumab vs Cetuximab Plus Chemotherapy After First Progression in KRAS Wild-Type Metastatic Colorectal Cancer: The UNICANCER PRODIGE18 Randomized Clinical Trial. JAMA Oncol. 2019, 5, 83–90, doi:10.1001/jamaoncol.2018.4465.
41. Shitara, K.; Yamanaka, T.; Denda, T.; Tsuji, Y.; Shinozaki, K.; Komatsu, Y.; Kobayashi, Y.; Furuse, J.; Okuda, H.; Asayama, M.; et al. REVERCE: A Randomized Phase II Study of Regorafenib Followed by Cetuximab versus the Reverse Sequence for Previously Treated Metastatic Colorectal Cancer Patients. Ann. Oncol. Off. J. Eur. Soc. Med. Oncol. 2019, 30, 259–265, doi:10.1093/annonc/mdy526.
42. Pietrantonio, F.; Lobefaro, R.; Antista, M.; Lonardi, S.; Raimondi, A.; Morano, F.; Mosconi, S.; Rimassa, L.; Murgioni, S.; Sartore-Bianchi, A.; et al. Capecitabine and Temozolomide versus FOLFIRI in RAS-Mutated, MGMT-Methylated Metastatic Colorectal Cancer. Clin. Cancer Res. Off. J. Am. Assoc. Cancer Res. 2020, 26, 1017–1024, doi:10.1158/1078-0432.CCR-19-3024.
43. Oki, E.; Emi, Y.; Yamanaka, T.; Uetake, H.; Muro, K.; Takahashi, T.; Nagasaka, T.; Hatano, E.; Ojima, H.; Manaka, D.; et al. Randomised Phase II Trial of MFOLFOX6 plus Bevacizumab versus MFOLFOX6 plus Cetuximab as First-Line Treatment for Colorectal Liver Metastasis (ATOM Trial). Br. J. Cancer 2019, 121, 222–229, doi:10.1038/s41416-019-0518-2.
44. Malka, D.; François, E.; Penault-Llorca, F.; Castan, F.; Bouché, O.; Bennouna, J.; Ghiringhelli, F.; de la Fouchardière, C.; Borg, C.; Samalin, E.; et al. FOLFOX Alone or Combined with Rilotumumab or Panitumumab as First-Line Treatment for Patients with Advanced Gastroesophageal Adenocarcinoma (PRODIGE 17-ACCORD 20-MEGA): A Randomised, Open-Label, Three-Arm Phase II Trial. Eur. J. Cancer Oxf. Engl. 1990 2019, 115, 97–106, doi:10.1016/j.ejca.2019.04.020.
45. Adenis, A.; Bennouna, J.; Etienne, P.L.; Bogart, E.; Francois, E.; Galais, M.P.; Ben Abdelghani, M.; Michel, P.; Metges, J.P.; Dahan, L.; et al. Continuation versus Discontinuation of First-Line Chemotherapy in Patients with Metastatic Squamous Cell Oesophageal Cancer: A Randomised Phase II Trial (E-DIS). Eur. J. Cancer Oxf. Engl. 1990 2019, 111, 12–20, doi:10.1016/j.ejca.2019.01.016.
46. Munemoto, Y.; Nakamura, M.; Takahashi, M.; Kotaka, M.; Kuroda, H.; Kato, T.; Minagawa, N.; Noura, S.; Fukunaga, M.; Kuramochi, H.; et al. SAPPHIRE: A Randomised Phase II Study of Planned Discontinuation or Continuous Treatment of Oxaliplatin after Six Cycles of Modified FOLFOX6 plus Panitumumab in Patients with Colorectal Cancer. Eur. J. Cancer Oxf. Engl. 1990 2019, 119, 158–167, doi:10.1016/j.ejca.2019.07.006.
47. Kobayashi, H.; Uetake, H.; Yasuno, M.; Sugihara, K. Effectiveness of Wound-Edge Protectors for Preventing Surgical Site Infections after Open Surgery for Colorectal Disease: A Prospective Cohort Study with Two Parallel Study Groups. Dig. Surg. 2019, 36, 83–88, doi:10.1159/000488214.
48. Hurwitz, H.I.; Tan, B.R.; Reeves, J.A.; Xiong, H.; Somer, B.; Lenz, H.-J.; Hochster, H.S.; Scappaticci, F.; Palma, J.F.; Price, R.; et al. Phase II Randomized Trial of Sequential or Concurrent FOLFOXIRI-Bevacizumab Versus FOLFOX-Bevacizumab for Metastatic Colorectal Cancer (STEAM). The oncologist 2019, 24, 921–932, doi:10.1634/theoncologist.2018-0344.

49. Fokas, E.; Allgäuer, M.; Polat, B.; Klautke, G.; Grabenbauer, G.G.; Fietkau, R.; Kuhnt, T.; Staib, L.; Brunner, T.; Grosu, A.-L.; et al. Randomized Phase II Trial of Chemoradiotherapy Plus Induction or Consolidation Chemotherapy as Total Neoadjuvant Therapy for Locally Advanced Rectal Cancer: CAO/ARO/AIO-12. J. Clin. Oncol. Off. J. Am. Soc. Clin. Oncol. 2019, 37, 3212–3222, doi:10.1200/JCO.19.00308.
50. Modest, D.P.; Martens, U.M.; Riera-Knorrenschild, J.; Greeve, J.; Florschütz, A.; Wessendorf, S.; Ettrich, T.; Kanzler, S.; Nörenberg, D.; Ricke, J.; et al. FOLFOXIRI Plus Panitumumab As First-Line Treatment of RAS Wild-Type Metastatic Colorectal Cancer: The Randomized, Open-Label, Phase II VOLFI Study (AIO KRK0109). J. Clin. Oncol. Off. J. Am. Soc. Clin. Oncol. 2019, 37, 3401–3411, doi:10.1200/JCO.19.01340.
51. Kim, C.; Chon, H.J.; Kim, J.H.; Jung, M.; Nam, C.M.; Kim, H.S.; Kang, B.; Chung, H.C.; Rha, S.Y. Randomised Phase II Trial Comparing Four Front-Line Doublets in Asian Patients with Metastatic Gastric Cancer. Eur. J. Cancer Oxf. Engl. 1990 2019, 112, 20–28, doi:10.1016/j.ejca.2018.11.029.
52. Yoshikawa, T.; Muro, K.; Shitara, K.; Oh, D.-Y.; Kang, Y.-K.; Chung, H.C.; Kudo, T.; Chin, K.; Kadowaki, S.; Hamamoto, Y.; et al. Effect of First-Line S-1 Plus Oxaliplatin With or Without Ramucirumab Followed by Paclitaxel Plus Ramucirumab on Advanced Gastric Cancer in East Asia: The Phase 2 RAINSTORM Randomized Clinical Trial. JAMA Netw. Open 2019, 2, e198243, doi:10.1001/jamanetworkopen.2019.8243.
53. Páez, D.; Tobeña, M.; Fernández-Plana, J.; Sebio, A.; Virgili, A.C.; Cirera, L.; Barnadas, A.; Riera, P.; Sullivan, I.; Salazar, J. Pharmacogenetic Clinical Randomised Phase II Trial to Evaluate the Efficacy and Safety of FOLFIRI with High-Dose Irinotecan (HD-FOLFIRI) in Metastatic Colorectal Cancer Patients According to Their UGT1A 1 Genotype. Br. J. Cancer 2019, 120, 190–195, doi:10.1038/s41416-018-0348-7.
54. McGregor, L.M.; Skrobanski, H.; Ritchie, M.; Berkman, L.; Miller, H.; Freeman, M.; Patel, N.; Morris, S.; Rees, C.; von Wagner, C. Using Specialist Screening Practitioners (SSPs) to Increase Uptake of Bowel Scope (Flexible Sigmoidoscopy) Screening: Results of a Feasibility Single-Stage Phase II Randomised Trial. BMJ Open 2019, 9, e023801, doi:10.1136/bmjopen-2018-023801.
55. Winther, S.B.; Liposits, G.; Skuladottir, H.; Hofsli, E.; Shah, C.-H.; Poulsen, L.Ø.; Ryg, J.; Osterlund, P.; Berglund, Å.; Qvortrup, C.; et al. Reduced-Dose Combination Chemotherapy (S-1 plus Oxaliplatin) versus Full-Dose Monotherapy (S-1) in Older Vulnerable Patients with Metastatic Colorectal Cancer (NORDIC9): A Randomised, Open-Label Phase 2 Trial. Lancet Gastroenterol. Hepatol. 2019, 4, 376–388, doi:10.1016/S2468-1253(19)30041-X.
56. Hamada, K.; Uedo, N.; Tonai, Y.; Arao, M.; Suzuki, S.; Iwatsubo, T.; Kato, M.; Shichijo, S.; Yamasaki, Y.; Matsuura, N.; et al. Efficacy of Vonoprazan in Prevention of Bleeding from Endoscopic Submucosal Dissection-Induced Gastric Ulcers: A Prospective Randomized Phase II Study. J. Gastroenterol. 2019, 54, 122–130, doi:10.1007/s00535-018-1487-6.
57. Kienle, D.L.; Dietrich, D.; Ribi, K.; Wicki, A.; Quagliata, L.; Winterhalder, R.C.; Koeberle, D.; Horber, D.; Bastian, S.; Kueng, M.; et al. Cetuximab Monotherapy and Cetuximab plus Capecitabine as First-Line Treatment in Older Patients with RAS- and BRAF Wild-Type Metastatic Colorectal Cancer. Results of the Multicenter Phase II Trial SAKK 41/10. J. Geriatr. Oncol. 2019, 10, 304–310, doi:10.1016/j.jgo.2018.11.011.

58. Parikh, A.R.; Lee, F.-C.; Yau, L.; Koh, H.; Knost, J.; Mitchell, E.P.; Bosanac, I.; Choong, N.; Scappaticci, F.; Mancao, C.; et al. MAVERICC, a Randomized, Biomarker-Stratified, Phase II Study of MFOLFOX6-Bevacizumab versus FOLFIRI-Bevacizumab as First-Line Chemotherapy in Metastatic Colorectal Cancer. Clin. Cancer Res. Off. J. Am. Assoc. Cancer Res. 2019, 25, 2988–2995, doi:10.1158/1078-0432.CCR-18-1221.
59. Yamazaki, K.; Ariyoshi, N.; Miyauchi, H.; Ohira, G.; Kaneya, N.; Yamamoto, K.; Arai, K.; Yamazaki, S.; Matsubara, H.; Suzuki, T.; et al. A Randomized Controlled, Open-Label Early Phase II Trial Comparing Incidence of FOLFIRI.3-Induced Diarrhoea between Hangeshashinto and Oral Alkalization in Japanese Patients with Colorectal Cancer. J. Clin. Pharm. Ther. 2019, 44, 946–951, doi:10.1111/jcpt.13020.
60. Suwa, Y.; Watanabe, J.; Ota, M.; Suzuki, S.; Suwa, H.; Watanabe, K.; Saito, S.; Nagamine, K.; Momiyama, M.; Ishibe, A.; et al. Randomized Phase II Trial of the Prophylactic Use of Celecoxib for the Prevention of Oxaliplatin-Related Peripheral Vascular Pain in Capeox (YCOG1205). Cancer Chemother. Pharmacol. 2019, 83, 419–424, doi:10.1007/s00280-018-3739-9.
61. Bang, Y.-J.; Kang, Y.-K.; Ng, M.; Chung, H.C.; Wainberg, Z.A.; Gendreau, S.; Chan, W.Y.; Xu, N.; Maslyar, D.; Meng, R.; et al. A Phase II, Randomised Study of MFOLFOX6 with or without the Akt Inhibitor Ipatasertib in Patients with Locally Advanced or Metastatic Gastric or Gastroesophageal Junction Cancer. Eur. J. Cancer Oxf. Engl. 1990 2019, 108, 17–24, doi:10.1016/j.ejca.2018.11.017.
62. Cleary, J.M.; Horick, N.K.; McCleary, N.J.; Abrams, T.A.; Yurgelun, M.B.; Azzoli, C.G.; Rubinson, D.A.; Brooks, G.A.; Chan, J.A.; Blaszkowsky, L.S.; et al. FOLFOX plus Ziv-Aflibercept or Placebo in First-Line Metastatic Esophagogastric Adenocarcinoma: A Double-Blind, Randomized, Multicenter Phase 2 Trial. Cancer 2019, 125, 2213–2221, doi:10.1002/cncr.32029.
63. Gorbunova, V.; Beck, J.T.; Hofheinz, R.-D.; Garcia-Alfonso, P.; Nechaeva, M.; Cubillo Gracian, A.; Mangel, L.; Elez Fernandez, E.; Deming, D.A.; Ramanathan, R.K.; et al. A Phase 2 Randomised Study of Veliparib plus FOLFIRI±bevacizumab versus Placebo plus FOLFIRI±bevacizumab in Metastatic Colorectal Cancer. Br. J. Cancer 2019, 120, 183–189, doi:10.1038/s41416-018-0343-z.
64. Bendell, J.C.; Sauri, T.; Gracián, A.C.; Alvarez, R.; López-López, C.; García-Alfonso, P.; Hussein, M.; Miron, M.-L.L.; Cervantes, A.; Montagut, C.; et al. The McCAVE Trial: Vanucizumab plus MFOLFOX-6 Versus Bevacizumab plus MFOLFOX-6 in Patients with Previously Untreated Metastatic Colorectal Carcinoma (MCRC). The oncologist 2020, 25, e451–e459, doi:10.1634/theoncologist.2019-0291.
