# Supplementary material for: How to Balance Prognostic Factors in Controlled Phase II Trials: Stratified Permuted Block Randomization or Minimization? An Analysis of Clinical Trials in Digestive Oncology
Source: Curr Oncol. 2024 Jun 17;31(6):3513–28. doi: 10.3390/curroncol31060259 (PMC11202503; doi:10.3390/curroncol31060259)
Supplement: Supplementary file 1 [file curroncol-31-00259-s001.zip › curroncol-3034724-supplementary/File S2.pdf]

Supplementary material S2

Table S1 : Summary table of the studies used for the simulations

Study name	FFCD9803	PRODIGE16	PRODIGE20	PRODIGE25	PRODIGE35	PRODIGE37
Primary tumor site	Gastric or cardial adenocarcinoma without linitis	Hepatocellular carcinoma	Colorectal	Colorectal	Pancreatic	Pancreatic
Number of patients randomized	136	78	102	117	276	127
Allocation method used	Minimization	Minimization	Minimization	Minimization	Minimization	Minimization
Number of stratification variables	4	3	3	4	3	3
Variables used for the stratification	1)institution<br>2) tumor site(cardia Y/N)<br>3) prior adj chemo (Y/N)<br>4) PS (0-1vs2)	1) diameter of the primary tumor (< 5 vs ≥ 5 cm)<br>2) presence of one nodule versus the presence of more than one nodule<br>3) investigating center	1) CT (FU monotherapy versus doublet)<br>2)primary tumor (resection vs no resect)<br>3) Spitzer QoL (0–3 vs 4–7 vs 8–10)	1) Center<br>2) age (< 75 vs > 75)<br>3) TS- 5'UTR polymorphism (genotype 2R2R-2R3R vs 3R3R, centrally determined)<br>4) number of metastatic sites (1 vs >1).	1) Center<br>2) Biliary stent: Yes versus No<br>3) Age: ≤ 65 yo vs> 65 yo	1) Center<br>2) PS 0 versus 1 versus 2<br>3)1 vs >1 metastatic site
Number of arms	3	2	2	2	3	2
Number of centres	41	17	Not taken into account (36)	33	52	36
Primary endpoint	ORR	Occurrence of severe bleeding or liver failure during the week following each TACE	composite co-primary end point : assessed 4 months after randomization, efficacy (tumor control and absence of decrease of the Spitzer QoL index)<br>and safety (absence of severe cardiovascular toxicities and unexpected hospitalization).	6-month progression-free survival rate	6-month progression-free survival rate	6-month progression-free survival rate
Secondary endpoints	Progression-Free Survival (PFS), OS (Overall Survival), safety, duration of hospital stay, and quality of life (QOL).	Toxicities, PFS and OS.	objective tumor response rate (ORR), PFS, OS, and tolerance	Safety, quality of life, overall survival, and the impact of TS-5'UTR polymorphism.	OS, PFS, best response rate, duration of disease control (DDC), safety, especially neurotoxicity, median dose intensity of oxaliplatin, quality of life (QOL), and second-line therapy	PFS, OS, safety and quality of life (QLQ-C30)

Table S2: Re-simulation of re-sampled real databases with centers. Impact on imbalance depending on the choice of method - Table results.

	PRODIGE 20 [16] n=102, 2 arms 36 centers not taken into account		PRODIGE 16 [15] n=78, 2 arms 17 centers		PRODIGE 25 [17] n=117, 2 arms 33 centers		PRODIGE 37 [19] n=127, 2 arms 36 centers		FFCD 9803 [14] n=136, 3 arms 41 centers		PRODIGE 35 [18] n=276, 3 arms 52 centers	
	Minimization N = 1,000	SPBR, N = 1,000	Minimization, N = 1,000	SPBR, N = 1,000	Minimization, N = 1,000	SPBR, N = 1,000	Minimization, N = 1,000	SPBR, N = 1,000	Minimization, N = 1,000	SPBR, N = 1,000	Minimization, N = 1,000	SPBR, N = 1,000
**Total Imbalance**												
Mean (SD)	1 (1.2)	2 (1.9)	1 (1.3)	4 (3.2)	1 (0.9)	7 (5.3)	2 (1.1)	7 (5.4)	1 (0.8)	9 (4.8)	2 (1.2)	12 (6.3)
Median	0.0	2.0	2.0	4.0	1.0	7.0	1.0	7.0	1.0	9.0	2.0	12.0
Q1 - Q3	0.0 - 2.0	0.0 - 4.0	0.0 - 2.0	2.0 - 6.0	1.0 - 1.0	3.0 - 11.0	1.0 - 3.0	3.0 - 11.0	1.0 - 2.0	6.0 - 12.0	0.0 - 2.0	8.0 - 16.0
Min - Max	0.0 - 6.0	0.0 - 10.0	0.0 - 6.0	0.0 - 16.0	1.0 - 7.0	1.0 - 25.0	1.0 - 7.0	1.0 - 29.0	1.0 - 6.0	1.0 - 29.0	0.0 - 6.0	0.0 - 49.0
**Marginal Imbalance**												
Mean (SD)	8 (3.2)	11 (4.1)	22 (4.5)	29 (6.8)	46 (6.2)	72 (14.5)	45 (6.0)	67 (10.9)	63 (5.6)	94 (14.1)	79 (6.4)	118 (13.2)
Median	8.0	10.0	22.0	28.0	46.0	70.0	45.0	67.0	63.0	92.0	78.5	117.0
Q1 - Q3	4.0 - 10.0	8.0 - 12.0	18.0 - 24.0	24.0 - 32.0	42.0 - 50.0	62.0 - 80.0	41.0 - 49.0	59.0 - 75.0	59.0 - 66.0	84.0 - 102.0	74.0 - 83.0	109.0 - 126.0
Min - Max	4.0 - 20.0	4.0 - 30.0	10.0 - 36.0	12.0 - 54.0	32.0 - 70.0	40.0 - 130.0	29.0 - 67.0	41.0 - 117.0	46.0 - 87.0	62.0 - 161.0	58.0 - 108.0	84.0 - 185.0
**Within Stratum Imbalance**												
Mean (SD)	16 (5.4)	7 (1.4)	32 (4.4)	24 (2.4)	83 (4.8)	73 (3.6)	86 (4.7)	78 (3.6)	88 (4.2)	81 (3.4)	152 (6.8)	129 (4.9)
Median	16.0	8.0	32.0	24.0	83.0	73.0	87.0	79.0	88.0	81.0	152.0	129.0
Q1 - Q3	12.0 - 20.0	6.0 - 8.0	28.0 - 34.0	22.0 - 26.0	79.0 - 85.0	71.0 - 75.0	83.0 - 89.0	75.0 - 81.0	85.0 - 91.0	79.0 - 83.0	147.0 - 156.0	126.0 - 132.0
Min - Max	6.0 - 38.0	6.0 - 10.0	20.0 - 46.0	20.0 - 32.0	67.0 - 97.0	63.0 - 85.0	75.0 - 101.0	69.0 - 89.0	75.0 - 103.0	71.0 - 92.0	131.0 - 172.0	116.0 - 144.0

Figure S1 : Re-simulation of re-sampled real databases without centers. Impact on imbalance depending on the choice of method.

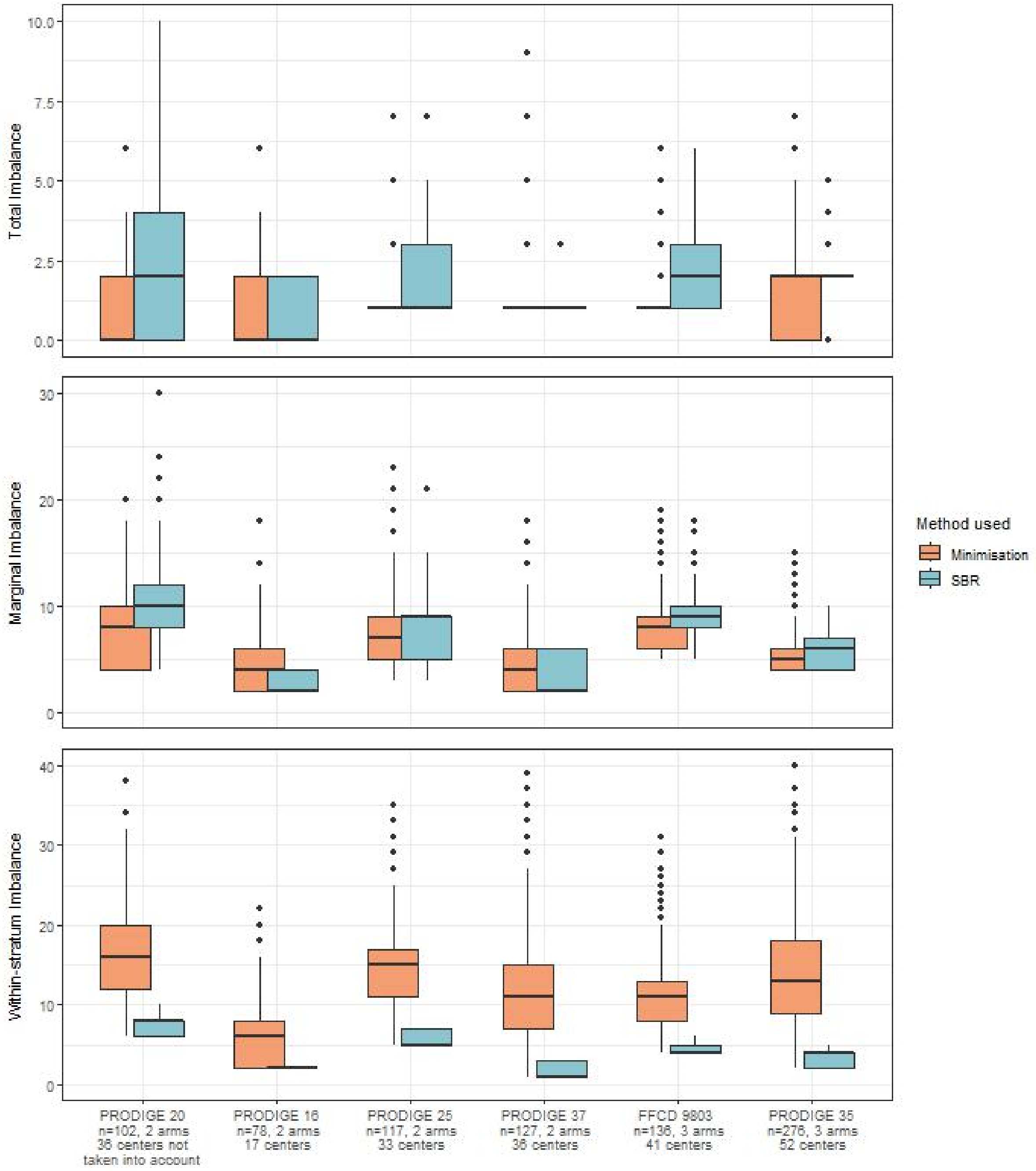

Table S3 : Re-simulation of re-sampled real databases without centers. Impact on imbalance depending on the choice of method – Table results

	PRODIGE 20 n=102, 2 arms		PRODIGE 16 n=78, 2 arms		PRODIGE 25 n=117, 2 arms		PRODIGE 37 n=127, 2 arms		FFCD 9803 n=136, 3 arms		PRODIGE 35 n=276, 3 arms	
	Minimization N = 1,000	SPBR, N = 1,000	Minimization, N = 1,000	SPBR, N = 1,000	Minimization, N = 1,000	SPBR, N = 1,000	Minimization, N = 1,000	SPBR, N = 1,000	Minimization, N = 1,000	SPBR, N = 1,000	Minimization, N = 1,000	SPBR, N = 1,000
**Total Imbalance**												
Mean (SD)	0.98 (1.20)	2.35 (1.91)	0.97 (1.20)	0.98 (1.00)	1.33 (0.83)	2.08 (1.35)	1.43 (0.95)	1.32 (0.74)	1.40 (0.80)	2.26 (1.19)	1.66 (1.19)	1.99 (1.13)
Median	0.00	2.00	0.00	0.00	1.00	1.00	1.00	1.00	1.00	2.00	2.00	2.00
Q1 - Q3	0.00 - 2.00	0.00 - 4.00	0.00 - 2.00	0.00 - 2.00	1.00 - 1.00	1.00 - 3.00	1.00 - 1.00	1.00 - 1.00	1.00 - 1.00	1.00 - 3.00	0.00 - 2.00	2.00 - 2.00
Min - Max	0.00 - 6.00	0.00 - 10.00	0.00 - 6.00	0.00 - 2.00	1.00 - 7.00	1.00 - 7.00	1.00 - 9.00	1.00 - 3.00	1.00 - 6.00	1.00 - 6.00	0.00 - 7.00	0.00 - 5.00
**Marginal Imbalance**												
Mean (SD)	7.87 (3.22)	10.58 (4.12)	3.86 (2.17)	2.98 (1.00)	6.99 (3.32)	8.31 (3.22)	5.02 (2.64)	3.32 (1.88)	7.95 (2.66)	9.33 (2.57)	5.58 (1.88)	5.55 (1.52)
Median	8.00	10.00	4.00	2.00	7.00	9.00	4.00	2.00	8.00	9.00	5.00	6.00
Q1 - Q3	4.00 - 10.00	8.00 - 12.00	2.00 - 6.00	2.00 - 4.00	5.00 - 9.00	5.00 - 9.00	2.00 - 6.00	2.00 - 6.00	6.00 - 9.00	8.00 - 10.00	4.00 - 6.00	4.00 - 7.00
Min - Max	4.00 - 20.00	4.00 - 30.00	2.00 - 18.00	2.00 - 4.00	3.00 - 23.00	3.00 - 21.00	2.00 - 18.00	2.00 - 6.00	5.00 - 19.00	5.00 - 18.00	4.00 - 15.00	4.00 - 10.00
**Within Stratum Imbalance**												
Mean (SD)	16.12 (5.42)	7.33 (1.36)	5.88 (3.83)	2.00 (0.00)	14.62 (5.37)	5.63 (0.93)	11.89 (6.54)	1.66 (0.94)	11.02 (4.36)	4.44 (0.58)	13.87 (6.65)	3.41 (1.07)
Median	16.00	8.00	6.00	2.00	15.00	5.00	11.00	1.00	11.00	4.00	13.00	4.00
Q1 - Q3	12.00 - 20.00	6.00 - 8.00	2.00 - 8.00	2.00 - 2.00	11.00 - 17.00	5.00 - 7.00	7.00 - 15.00	1.00 - 3.00	8.00 - 13.00	4.00 - 5.00	9.00 - 18.00	2.00 - 4.00
Min - Max	6.00 - 38.00	6.00 - 10.00	2.00 - 22.00	2.00 - 2.00	5.00 - 35.00	5.00 - 7.00	1.00 - 39.00	1.00 - 3.00	4.00 - 31.00	4.00 - 6.00	2.00 - 40.00	2.00 - 5.00

*Figure S2 : Re-simulation of re-sampled real PRODIGE 35 database (A) and PRODIGE 37 (B): relative difference between the efficacy concluded in the original clinical trial (6-month PFS) and the estimated efficacy in each of the 1000 simulated data-sets according to SPBR or minimization allocation treatment arm method. The boxes corresponds to the 25th-75th percentiles relative difference, central segment corresponds to the median relative difference, the whiskers corresponds to the 1.5*standard deviation from the mean relative and dots represents the mean.*

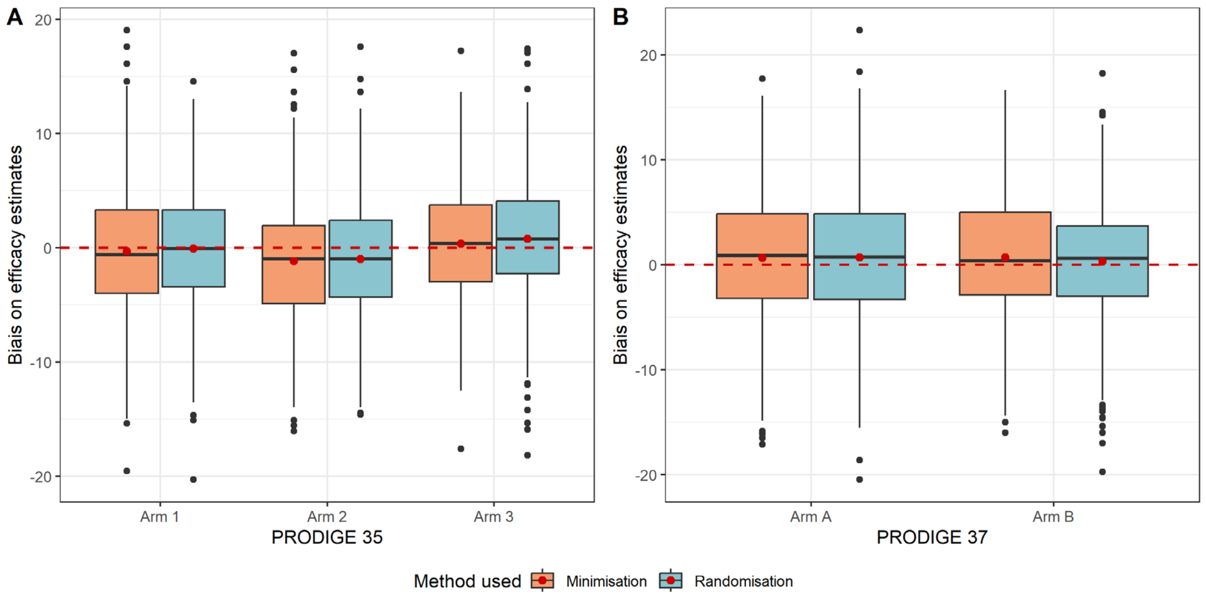

Table S4 : Results table of the re-simulation of re-sampled real PRODIGE 35 database and PRODIGE 37: relative difference between the efficacy concluded in the original clinical trial (6-month PFS) and the estimated efficacy in each of the 1000 simulated data-sets according to SPBR or minimization allocation treatment arm method

		**6-month PFS published**	**Resimulated with minimization**	**Bias (published-simulated)**	**90 % CI 5%-95% quantile**	**Resimulated with SPBR**	**Bias (published-simulated)**	**90 % CI 5%-95% quantile**
PRODIGE 35	ARM 1	47.1 %	47.4	-0.3	38.2 56.2	47.2	-0.1	38.2 56.2
	ARM 2	42.9% (90% CI, 34.3 to 51.4)	44.0	-1.2	35.5 52.3	43.9	-1.0	35.6 52.3
	ARM 3	34.1% (90% CI, 25.7 to 43.3)	33.7	0.4	25.8 42.0	33.3	0.8	25.3 41.6
PRPDIGE 37	ARM A	45.2% [one-sided 95% CI: 34.3-56.4]	44.5	0.7	32.8 56.7	44.5	0.7	32.8 56.5
	ARM B	23.3% in arm B [one-sided 95% CI: 14.3-32.3]	22.6	0.7	13.1 33.3	23.0	0.3	12.9 33.9
